# Competencies required by sonographers teaching ultrasound interprofessionally: a Delphi consensus study

**DOI:** 10.1186/s12909-024-05933-x

**Published:** 2024-09-05

**Authors:** Carolynne J. Cormack, Jessie Childs, Fiona Kent

**Affiliations:** 1 Faculty of Medicine Nursing and Health Sciences, Monash University, Wellington Rd, Clayton, VIC 3800 Australia; 2 Faculty of Allied Health and Human Performance, University of South Australia, Adelaide, Australia; 3 Royal College of Surgeons in Ireland (RCSI), Health Professions Education Centre, Dublin, Ireland

**Keywords:** Ultrasonography, Ultrasound, Point-of-care ultrasound, Sonographer, Training, Education, Competency

## Abstract

**Background:**

Clinicians from multiple professional backgrounds are increasingly using point-of-care ultrasound in clinical practice. Performing ultrasound is a complex skill, and training is required to ensure competency and patient safety. There is a lack of skilled trainers within health professions to meet this increasing educational demand. The role of sonographers in educating other health professionals in point-of-care ultrasound has not yet been well defined. Sonographers can provide ultrasound education interprofessionally, if equipped with appropriate clinical knowledge and educational skills.

**Methods:**

A Delphi consensus study was conducted to define the knowledge, skills and attributes required of sonographers teaching point-of-care ultrasound to other health professionals in Australia and New Zealand. Health professionals with subject matter expertise in the leadership, facilitation, and delivery of ultrasound education by sonographers were invited to participate.

**Results:**

There were 72 expert participants in survey round one, and 49 in round two. Participants included physicians, sonographers, and other health professionals. Consensus was reached on 31 competency items for sonographers teaching ultrasound interprofessionally, with agreement of greater than 94% reached by participants.

**Conclusions:**

This consensus study has defined the knowledge, skills and attitudes required for sonographer competence in point-of-care ultrasound education. This is an important step to developing a training pathway for sonographers engaging in this emerging area.

## Background

Rapid advances in technology and portability over the last two decades have resulted in many health professions adopting ultrasound as a clinical tool [1–4]. This is commonly referred to as ‘point-of-care ultrasound’ but is also known by other terms including ‘focused’, ‘bedside’, ‘hand-held’, ‘critical care’ and ‘emergency’ ultrasound. Importantly, point-of-care ultrasound (POCUS) differs from the comprehensive ultrasound scans performed by imaging professionals. POCUS describes a clinically focused ultrasound examination performed at the patient ‘point of care’ by the treating clinician [5]. It may be used as part of bedside physical examination, triage, diagnosis, surveillance or for guidance of procedures [1–5].

Performing ultrasound is a complex psychomotor skillset that requires specific training and deliberate practice [6–9]. Competence in performing ultrasound is developed via clinical practice, supervision and feedback. There is need for increased ultrasound training amongst Australasian health professions [10–12]. A common challenge identified in POCUS is the lack of expert trainers available to support the development of ultrasound skills in the clinical environment. Similar challenges have been reported in the United Kingdom, Canada and United States [13–15]. In Australasia, sonographers are engaging in POCUS education and have been recognised for their expertise in providing hands-on interprofessional ultrasound skills training [16–19]. A recent survey found 20% of Australasian emergency departments report accessing sonographer educators for ultrasound education [12]. However, this new interprofessional education role is not yet well defined and sonographers are an under-recognised resource to help address the POCUS education gaps.

Sonographers are post-graduate qualified allied health professionals in Australia and New Zealand. Sonographers are registered with the Australian Sonographer Accreditation Registry (ASAR) or New Zealand Medical Radiation Technologists Board (NZMRTB). Sonographer post-graduate training includes the theory of ultrasound physics, sonographic anatomy and pathology; and certification requires more than 2000 h of supervised clinical practice. Sonographers are experts in ultrasound imaging, with a clinical role centred on providing comprehensive ultrasound imaging within medical imaging departments. A recently revised Australian sonographer competency framework includes education as part of a clinical sonographer role, with responsibility for the development of the complex psychomotor, image interpretation and critical thinking skills of trainee sonographers [16, 20]. However, this education is typically oriented to teaching ultrasound imaging to sonography trainees and there is no expectation for formal clinical education qualifications [21]. Providing POCUS education interprofessionally requires additional knowledge of clinical contexts, scope of practice, professional frameworks and educational strategies. By clarifying the additional competencies required for sonographer educators to teach POCUS, a training pathway can be established. The research question posed was: *what knowledge*,* skills and attributes are required by sonographer educators training other health professions in point of care ultrasound?*

## Methods

### Research design

A modified Delphi consensus study was conducted to elicit expert opinion on the competencies (knowledge, skills and attributes) required for sonographers teaching ultrasound (POCUS) interprofessionally. The Delphi method provides a means of answering a research question by seeking consensus across a group of subject matter experts. Experts were considered in this study to be ‘informed individuals, specialists, and those with knowledge about a specific subject’ [22]. The Delphi method is an iterative process allowing for review and modification of opinions based on anonymised feedback of results in each survey round, with the goal of reaching consensus [22, 23]. Consensus was determined a priori to be achieved at 75% agreement or greater on each statement, as per Delphi method recommendations [24].

### Participants and recruitment

Health professionals involved in the leadership, facilitation and delivery of ultrasound education in Australia and New Zealand were sought. Recruitment of sonographers, physicians, nurses and allied health professionals with subject matter expertise was made via professional organisations. Invitation to participate in the research study was disseminated by email or newsletter to members of the Australasian Society of Ultrasound in Medicine (ASUM), Australasian Sonographers Association (ASA) and Emergency Medicine Ultrasound Groups (EMUGs) networks. Online advertising was also adopted using social media (LinkdIn, Facebook, X Twitter) and snowballing was encouraged.

Informed consent was obtained for round one via a research Explanatory Statement and acknowledgement of a ‘consent to participate’ field at the start of the electronic survey. At the end of survey round one, participants were invited to continue participation in round two by providing an email address for the second survey to be sent. The first survey was active for eight weeks and closed September 30, 2023. After these results were analysed and condensed, the second survey was active for four weeks and closed October 30, 2023. Reminder emails were sent to second round participants one week prior to closing. Although three survey rounds were initially planned, only two rounds were required due to the pre-determined consensus being achieved.

### Inclusion and exclusion criteria

Inclusion and exclusion criteria, and a working definition of POCUS education was communicated via the Explanatory Statement. Participants were required to have experience in the delivery, leadership or facilitation of POCUS education by sonographers, in Australia or New Zealand, within the last ten years. POCUS was defined as ultrasound scans performed and interpreted by clinicians at the patient ‘point of care’ (i.e. scans performed in emergency departments, intensive care units, maternity clinics, wards, community facilities, or during transit by air/road ambulance). POCUS education *included* the teaching and training of non-imaging health professionals using ultrasound (i.e. physicians, midwives, nurses, nurse practitioners, paramedics, physiotherapists or other qualified health professionals). It *included* all ultrasound scans performed by non-imaging health professionals. This definition of POCUS education *excluded* any ultrasound teaching of imaging professionals as this is not POCUS education (i.e. radiologists, cardiologists, radiology/cardiology trainees, sonographers, echocardiographers or sonographer trainees). It also *excluded* any portable or mobile ultrasound scans performed and reported by imaging professionals, as this is not POCUS.

### Instrument

The first round Delphi survey items were pooled from multiple sources with the potential to inform the interprofessional role of sonographer educators in POCUS. These sources were: Australian Professional Competency Framework for Sonographers; Australasian College for Emergency Medicine (ACEM) Sonographer Educators in Emergency Departments (SEED) definition and position description documents; and Australasian Society of Ultrasound in Medicine (ASUM) Certificate in Clinician Performed Ultrasound (CCPU) regulations [16–19]. A draft survey was piloted by the authors (CC, JC, FK), with modifications made to the synthesis of items and organisation of the sequence for logic.

The first survey included demographic questions about participant health professions, roles, education qualifications, ultrasound qualifications and POCUS experience. Participants were then asked to indicate their level of agreement with an exhaustive list of 38 initial competencies (knowledge, skills and attributes) required by sonographer educators teaching across the health professions. Items were rated on a 4-point Likert scale: ‘agree’, ‘neutral’, ‘disagree’, or ‘unable to comment’ (when participants lacked specific experience to provide opinion on that item). Open-ended questions were asked at the end of each section to capture any additional knowledge, skills and attributes and provide comment on the wording of any items.

The second survey presented the results of survey round one, and a refined competency list of 31 items for participants to rate on the same 4-point Likert scale. Modifications that were made based on round one feedback were explained to participants. This refined list of items was grouped into professional, clinical and educational domains in the second survey. Other modifications included removal of three items that did not achieve consensus, merging of 10 items describing a similar construct, the addition of one potential new item and minor modifications to the wording of several items for greater clarity.

### Data collection and analysis

Electronic survey platform Qualtrics Insight (Qualtrics XM, Provo, Utah, USA) was used for data collection. Data was exported from Qualtrics to Microsoft Xcel for analysis. Reporting adhered to ACCORD (Accurate Consensus Reporting Document) guidelines [25].

### Research team

The research team was composed of a sonographer with expertise in POCUS education (CC), a sonographer with expertise in ultrasound education and research (JC), and physiotherapist with expertise in health professional educational research (FK).

## Results

### Participant demographics

There were 72 round one participants, following the removal of incomplete entries from an initial 106 responses. There were 49 round two participants. Survey round one participants included 37 sonographers (51%), 31 physicians (43%), 3 nurses (4%) and 1 paramedic (1%). Participants were Australian (87%) and Aotearoan New Zealander (13%). Participants reported involvement in ultrasound education across all regions: New Zealand (13%), New South Wales (24%), Victoria (24%), Western Australia (6%), South Australia (4%), Queensland (1%), Tasmania (1%) and multiple states/territories (28%). Respondents had experience working in diverse geographical contexts, including multiple metropolitan, regional and remote locations (43%); metropolitan only (46%); and regional or remote contexts only (11%).

### Participant qualifications and experience

**Fig. 1:**
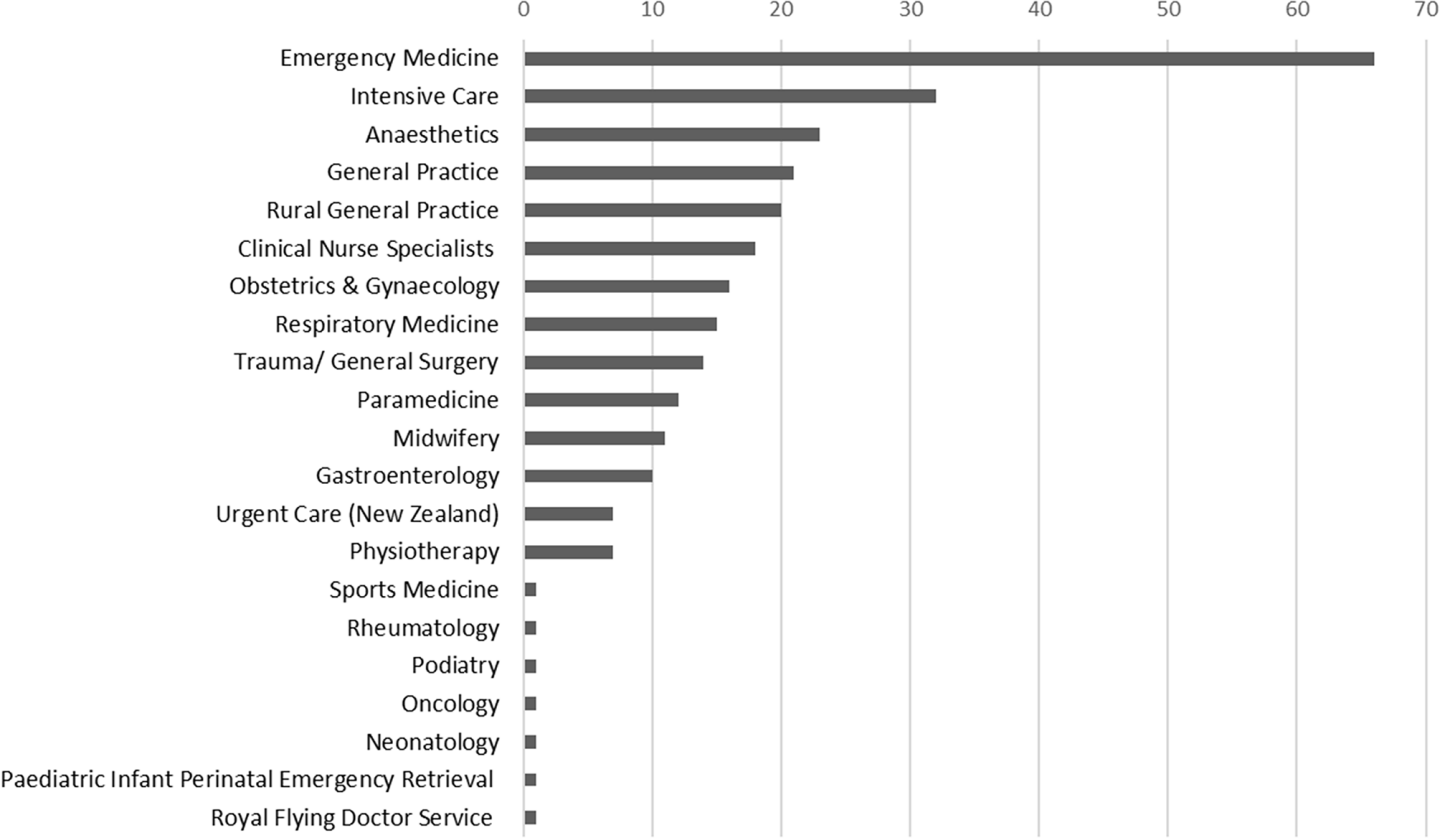
Participant teaching experience by health professional specialties

Participants had varied ultrasound and educational qualifications and experience (Table 1). Ultrasound teaching experience included public/ private hospitals (94%), private education companies (40%) and universities (31%). Experience teaching POCUS across many medical disciplines was reported including: Emergency Medicine (92%), Intensive Care (44%), Anaesthetics (32%), General Practice (29%) and Rural General Practice (28%) (Fig. 1). Professional ultrasound-related affiliations included: ASUM Australasian Society of Ultrasound in Medicine (57%), ASA Australasian Sonographers Association (36%), WFUMB World Federation for Ultrasound in Medicine and Biology (18%), ASE American Society of Echocardiography (10%), CSANZ Cardiac Society of Australia and New Zealand (7%), and ISUOG International Society of Ultrasound in Obstetrics and Gynaecology (4%).

Amongst the 37 sonographers participating, all held post graduate ultrasound qualifications, including Graduate Diploma of Medical Ultrasound or equivalent (100%), Masters of Medical Ultrasound (22%) and Doctor of Philosophy (3%). Within this sonographer cohort, 84% reported more than 10 years clinical ultrasound experience, and 57% more than 20 years. Additionally, 46% reported more than five years of experience teaching POCUS, and 24% more than 10 years. By speciality, 73% had qualifications and clinical expertise in general, obstetric, vascular, musculoskeletal and paediatric ultrasound; and 27% had cardiac qualifications. They reported accreditation with relevant professional bodies (100%) and other ultrasound related professional affiliations (97%). Sonographers also reported senior clinical and educational roles that included: senior sonographer (70%), tutor sonographer (57%), POCUS educator (59%), university academic (59%) and chief sonographer (38%).

Amongst the 31 physicians participating, there were specialists in Emergency Medicine (94%), Intensive Care (3%) and General Practice (3%). Of this medical cohort, 52% reported more than 10 years POCUS teaching experience. Participating physicians also reported holding senior clinical leadership and educational roles, including Clinical Lead/ Director of Emergency Ultrasound (71%), Supervisor/ Director of Training (29%) and Director/ Deputy Director of Department (19%).

**Table 1:** Participant ultrasound qualifications and experience

ULTRASOUND QUALIFICATIONS	SONOGRAPHER (*n* = 37)	PHYSICIAN, NURSE, ALLIED HEALTH (*n* = 35)
Accredited Medical Sonographer - Australasian Sonographer Accreditation Registry (ASAR), New Zealand Medical Radiation Technologists Board (NZMRTB)	37 (100%)	N/A
University Graduate Diploma Medical Ultrasound (GDMU), Graduate Certificate/Diploma Clinical Ultrasound (GCCU, GDCU)	28 (76%)	6 (17%)
ASUM Diploma Medical Ultrasound (DMU), Diploma Diagnostic Ultrasound (DDU)	9 (24%)	7 (20%)
ASUM Certificate Clinician Performed Ultrasound (CCPU) or equivalent	N/A	15 (43%)
University Master Medical Ultrasound (MMU), Master Clinical Ultrasound (MCU)	8 (22%)	2 (6%)
Doctor of Philosophy (PhD) clinical ultrasound or education thesis	2 (5%)	0
Currently undertaking ultrasound qualifications	0	8 (23%)
**EDUCATIONAL QUALIFICATIONS**		
Certificate IV Training and Assessment	4 (11%)	0
University Graduate Certificate/ Diploma Health Professions Education/ Clinical Simulation/ Education and Training	3 (8%)	4 (11%)
Master Health Professions Education/ Clinical Simulation	3 (8%)	0
**ULTRASOUND TEACHING CONTEXTS**		
Public or private teaching hospitals	34 (92%)	34 (97%)
Urgent care clinics (NZ)	3 (8%)	2 (6%)
Community health clinic/ private medical clinics	11 (30%)	1 (3%)
Mobile/ retrieval / air or road ambulance services	6 (16%)	5 (14%)
University based education	14 (38%)	8 (23%)
Private ultrasound education	15 (41%)	14 (40%)
Ultrasound equipment vendor	9 (24%)	4 (11%)

### Delphi survey outcomes

Consensus on the competencies (knowledge, skills and attitudes) for sonographer educators was reached in two Delphi survey rounds (Table 2). Round one initially proposed 38 competency items. Round two proposed 31 refined competency items grouped into three domains: professional, clinical and educational. The professional domain had eight items relating to professional conduct, excellence and communication. The clinical domain had 11 items relating to clinical knowledge, diagnostic judgement and patient care. The educational domain had 12 items relating to best practice in clinical teaching, learner engagement and competency standards. Consensus was achieved on 31/31 (100%) of the round two refined items, with high levels of agreement on all final items (between 94 and 100%).

**Table 2:** Delphi survey outcomes - sonographer educator competencies

COMPETENCY ITEMS (KNOWLEDGE, SKILLS, ATTITUDES) % AGREEMENT (*n* = 49)		
**PROFESSIONAL DOMAIN**		
1	Demonstrates a commitment to high-quality care and excellence in all aspects of practice	48 (98%)
2	Demonstrates patience, accountability, humility, open-mindedness, resilience, and respect for others	49 (100%)
3	Practices in an ethical and professional manner, consistent with relevant legislative and professional standards, including respecting autonomy, dignity of the individual, causing no harm, patient confidentiality, valid consent, duty of care and occupational health and safety	49 (100%)
4	Communicates using a patient-centred approach that encourages patient trust and autonomy, and is empathic, respectful, compassionate, non-judgemental and sensitive to culture	48 (98%)
5	Communicates respectfully with colleagues to address concerns, clarify expectations and to give or receive constructive criticism	49 (100%)
6	Participates in teamwork and maintains respectful collaborative relationships with healthcare teams, departments and organisations	49 (100%)
7	Demonstrates flexibility and adaptability, and is capable of dealing with difficult clinical scenarios	47 (96%)
8	Lifelong learner engaged in reflective practice, evaluating personal strengths or limitations to identify learning areas and adapt professional practice	49 (100%)
**CLINICAL DOMAIN**		
9	Experienced sonographer with a broad knowledge of general or cardiac ultrasound, including technical, clinical and teaching skills and minimum five years clinical experience	48 (98%)
10	Awareness of the evolving role of POCUS in the health professions, including understanding of the clinical context, indications and role of ultrasound in the relevant specialty	48 (98%)
11	Understands distinctions between a comprehensive imaging pathology-based paradigm, and a clinically focused symptom-based paradigm utilised in POCUS	49 (100%)
12	Employs critical thinking to resolve clinical challenges, incorporating appraisal and synthesis of research literature, and clinical experience to inform clinical reasoning	49 (100%)
13	Applies clinical experience, knowledge and judgement regarding technical or diagnostic limitations of a scan; understands the role of other imaging modalities and imaging pathways	48 (98%)
14	Exercises appropriate levels of autonomy and professional judgement in sonographic practice; understands scope of practice and risk minimisation; knows when to escalate to other health professionals and understands governance structures to address concerns	46 (94%)
15	Ensures patient safety, dignity and care is maintained when learners are involved	47 (96%)
16	Ensures appropriate documentation of scans that accurately represent any pathology present and follow organisational protocols (e.g. still images/cine loops, written reports, patient notes, oral summary)	49 (100%)
17	Ensures the communication of significant findings are accurate and timely for other health professionals to make informed clinical decisions	48 (98%)
18	Knowledge of POCUS scan protocols and principles of procedural guidance as defined by relevant professional organisations e.g. eFAST, LUNG, AAA, CARDIAC	46 (94%)
19	Skilled in optimising POCUS equipment to deliver best clinical results; assessing suitability for required examinations/procedures; advising on quality assurance and maintenance; recognising and reporting equipment faults	49 (100%)
**EDUCATIONAL DOMAIN**		
20	Knowledge of professional standards, guidelines, curricula and assessment tools endorsed by relevant professional organisations e.g. ASUM, ASA, ACEM, CICM, RANZCOG, WFUMB	48 (98%)
21	Knowledge of best practice in teaching, clinical supervision, mentorship, peer review, feedback and assessment techniques	48 (98%)
22	Self-motivated and enthusiastic ultrasound educator	47 (96%)
23	Able to provide practical psychomotor skills training and supervision of staff performing POCUS, working closely with other educators to ensure best educational outcomes	49 (100%)
24	Demonstrates the skills required to supervise health professionals in the work environment, including assessment and appropriate feedback (verbal/written) for the learner and education provider	48 (98%)
25	Identifies ongoing professional learning needs and opportunities to promote best practice, developing training tools, useful teaching resources, and prioritises learning opportunities	49 (100%)
26	Aligns learning goals to the development of competence and improvement of clinical practice quality; shares interesting cases, new research, and addresses gaps in the knowledge base	49 (100%)
27	Promotes a safe learning environment, recognises the learning needs of individuals and adapts approach to address issues hindering learning	49 (100%)
28	Communicates effectively with other healthcare professionals using communication strategies to facilitate interprofessional learning	47 (96%)
29	Able to describe and apply knowledge of anatomy, variants, embryology, pathophysiology, haemodynamics, ultrasound physics, instrumentation, artefacts and bioeffects to other health professionals	48 (98%)
30	Able to explain and discuss potential diagnostic pitfalls in ultrasound to other health professionals (e.g. ultrasound artefacts, equipment settings, measurements, interpretive errors)	48 (98%)
31	Able to assist in development of POCUS education programs as agreed (e.g. logbook case review, educational presentations, teaching materials, scan protocols, clinical audits, research)	47 (96%)

## Discussion

Current gaps in POCUS education have the potential to be met by sonographers who are suitably equipped. This Delphi study has defined the knowledge, skills and attitudes required for sonographer competence in POCUS education. Clarification of the competencies required of POCUS educators is an important step toward developing a training pathway for sonographers. Quality healthcare requires competent educators in medicine, nursing, and allied health to train other healthcare workers at all stages of their professional development [26]. Further investigation of the transitions and challenges experienced by sonographers moving into interprofessional POCUS education roles has been explored in a second phase of this study.

Sonographer engagement in interprofessional education differs from intraprofessional ultrasound education in several respects. Teaching interprofessionally requires knowledge of the professional frameworks, competency standards and educational needs of other health professions. It requires an understanding of the clinical scopes of practice and patient care pathways relevant to other health professions. Teaching in different clinical environments can also be challenging [27, 28]. POCUS training often occurs in fast-paced clinical environments and involves ‘teaching on the run’, with limited time for preparatory instruction and learner feedback. This requires different teaching strategies for sonographer educators to achieve necessary learning outcomes. The immediate integration of scan findings into patient management is another aspect of POCUS that requires advanced clinical reasoning.

Multiple competency items identified in this study related to communication and interpersonal skills, as has been reported in the broader medical education literature [29]. Success as a POCUS educator requires additional skill in building respectful, collaborative relationships for interprofessional teaching. The affective attributes of enthusiasm and dedication identified in this study replicates recognised characteristics of good clinical teachers [29]. Additional skill and sensitivity in accommodating different learner needs was articulated, as was the need for flexibility and judgement when teaching in the clinical environment. Breadth of clinical knowledge was also emphasised, with the expectation that sonographer educators be experienced and well informed about the specific ultrasound protocols and competency requirements of other professions.

This study has outlined further development needed for sonographers in POCUS education roles. Professional regulation and career advancement for sonographers in Australasia is less developed than in the United Kingdom, where there is autonomy in reporting and established pathways for recognition of advanced and consultant practice [30–32]. Role extension, extended practice, advanced skills and advanced practice are all terms that have been used to describe the evolving scope of allied health roles. It is recognised that increasing the knowledge, skills, and capabilities of healthcare staff increases quality and safety outcomes, as well as workforce retention [33, 34]. Australian and New Zealand government agencies responsible for the allied health workforce support the principles of professional growth and innovation to keep pace with emerging technologies and changing clinical practice [35, 36]. The Australian Government 10-year health plan includes goals to ‘realign workforce education…to reflect advances in models of care’, however progress to formalise extended roles for allied health professions in Australia has been slow, and sonographers have yet to realise their full professional potential [35, 37, 38].

As POCUS technology evolves, so too are technology-based educational tools to support learning. Resources such as digital learning platforms, ultrasound simulators using virtual or augmented reality, and artificial intelligence are increasingly playing a role in ultrasound education. These tools will be valuable for establishing foundational knowledge, although will not replace the need for learning in the clinical environment with educators to instruct, supervise, review cases, provide feedback and assess competency. A recognised challenge in POCUS education is the lack of skilled educators available [10–15].

### Strengths and limitations

This consensus study sought to gain the perspective of subject matter experts from Australia and New Zealand, to determine the competencies required for sonographer educators teaching POCUS interprofessionally. A broad demographic mix of participants, with significant clinical expertise in ultrasound education, completed two survey rounds to consensus [23]. The final list of 31 competency items may be perceived as somewhat overwhelming to new POCUS educators, however it augments existing sonographer competency standards, and a comprehensive list is useful to facilitate development of a targeted training pathway.

## Conclusion

This consensus study has defined the knowledge, skills and attitudes required for sonographer competence in providing interprofessional ultrasound education. High levels of agreement were reached by participants with expertise in ultrasound education in Australia and New Zealand. Further investigation of the challenges and transitions for sonographers engaging in POCUS education is planned in a second study phase, with the goal to develop continuing education for sonographers in this emerging area.

## Data Availability

The data that support the findings of this study are available on request from the corresponding author, CC. The data are not publicly available due to participant privacy protection.

## Abbreviations

ACEM: Australasian College for Emergency Medicine
ASA: Australasian Sonographers Association
ASE: American Society of Echocardiography
ASUM: Australasian Society of Ultrasound in Medicine
ASAR: Australian Sonographer Accreditation Registry
CCPU: Certificate of Clinician Performed Ultrasound
CSANZ: Cardiac Society of Australia and New Zealand
DDU: Diploma of Diagnostic Ultrasound
EMUGs: Emergency Medicine Ultrasound Groups
ISUOG: International Society of Ultrasound in Obstetrics and Gynaecology
NZMRTB: New Zealand Medical Radiation Technologists Board
POCUS: Point of care ultrasound
SEED: Sonographer educator in emergency department
WFUMB: World Federation for Ultrasound in Medicine and Biology
